# Ginsenoside Rg1 Suppresses Type 2 PRRSV Infection via NF-κB Signaling Pathway In Vitro, and Provides Partial Protection against HP-PRRSV in Piglet

**DOI:** 10.3390/v11111045

**Published:** 2019-11-10

**Authors:** Zhi-qing Yu, He-you Yi, Jun Ma, Ying-fang Wei, Meng-kai Cai, Qi Li, Chen-xiao Qin, Yong-jie Chen, Xiao-liang Han, Ru-ting Zhong, Yao Chen, Guan Liang, Qiwei Deng, Kegong Tian, Heng Wang, Gui-hong Zhang

**Affiliations:** 1 College of Veterinary Medicine, South China Agricultural University, Guangzhou 510462, China; zhiqingyu@stu.scau.edu.cn (Z.-q.Y.); heyouyi@stu.scau.edu.cn (H.-y.Y.); 18814116634@163.com (J.M.); yingfangwe@163.com (Y.-f.W.); caimengkai@126.com (M.-k.C.); Qili@126.com (Q.L.); qinchenxiaol@163.com (C.-x.Q.); XiaoliangHan@scau.edu.cn (X.-l.H.); RutingZhong@scau.edu.cn (R.-t.Z.); Liangguan@scau.edu.cn (G.L.); dengqiwei@scau.edu.cn (Q.D.); 2 Key Laboratory of Zoonosis Prevention and Control of Guangdong Province, Guangzhou 510462, China; vetcyj@163.com; 3 School of Life Science and Engineering, Foshan University, Foshan 528225, China; chenyao1991scau@foxmail.com; 4 College of Animal Science and Veterinary Medicine, Henan Agricultural University, Zhengzhou 450002, China; 123456@163.com; 5 OIE Reference Laboratory for PRRS in China, China Animal Disease Control Center, Beijing 100125, China

**Keywords:** porcine reproductive and respiratory syndrome virus, ginsenoside Rg1, antiviral activity, pro-inflammatory factor, NF-κB signaling pathway

## Abstract

Porcine reproductive and respiratory syndrome virus (PRRSV) is a huge threat to the modern pig industry, and current vaccine prevention strategies could not provide full protection against it. Therefore, exploring new anti-PRRSV strategies is urgently needed. Ginsenoside Rg1, derived from ginseng and notoginseng, is shown to exert anti-inflammatory, neuronal apoptosis-suppressing and anti-oxidant effects. Here we demonstrate Rg1-inhibited PRRSV infection both in Marc-145 cells and porcine alveolar macrophages (PAMs) in a dose-dependent manner. Rg1 treatment affected multiple steps of the PRRSV lifecycle, including virus attachment, replication and release at concentrations of 10 or 50 µM. Meanwhile, Rg1 exhibited broad inhibitory activities against Type 2 PRRSV, including highly pathogenic PRRSV (HP-PRRSV) XH-GD and JXA1, NADC-30-like strain HNLY and classical strain VR2332. Mechanistically, Rg1 reduced mRNA levels of the pro-inflammatory cytokines, including IL-1β, IL-8, IL-6 and TNF-α, and decreased NF-κB signaling activation triggered by PRRSV infection. Furthermore, 4-week old piglets intramuscularly treated with Rg1 after being challenged with the HP-PRRSV JXA1 strain display moderate lung injury, decreased viral load in serum and tissues, and an improved survival rate. Collectively, our study provides research basis and supportive clinical data for using Ginsenoside Rg1 in PRRSV therapies in swine.

## 1. Introduction

Porcine reproductive and respiratory syndrome (PRRS), characterized by respiratory distress, reproductive failure in pregnant sows and high mortality in piglets [1], is one of the most epidemic porcine infectious diseases that cause huge economic losses in the worldwide pig industry. Porcine reproductive and respiratory syndrome virus (PRRSV) is a positive-sense, single-stranded RNA virus, and it belongs to the Arteriviridae family [2]. PRRSV is divided into two genotypes, including European strains (type 1) and North American strains (type 2) [3]. Type 2 PRRSV is dominant in China for decades, and it is further classified into nine lineages based on the nucleotide sequence of the ORF5 gene [4,5].

Marc-145 cells, purchased from the American Type Culture Collection (ATCC, Manassas, Virginia, USA) and saved in our lab, were cultured in Dulbecco’s Minimum Essential Medium (DMEM, Biological Industries, Kibbutz Beit Haemek, Israel), supplemented with 10% fetal bovine serum (FBS, Biological Industries, Kibbutz Beit Haemek, Israel) at 37 °C with 5% CO_2_. Porcine alveolar macrophages (PAMs) were collected from the fresh lungs of 4-week-old Large-White piglets, which were free of the PRRSV and anti-PRRSV antibody, and prepared as previously described [6]. PAMs were grown in RPMI 1640 (Gibco, UT, USA) which contains 10% FBS and 100 IU/mL penicillin and 100 μg/mL streptomycin.

In recent decades, the emergence of a novel PRRSV strain and worldwide transmission attracted increasing attention [7,8,9,10,11]. Current major preventive strategies focus on the vaccine application. However, the poorly studied immunosuppression of pigs to PRRSV infection, virus evolution, multiple-recombination event between wild-type strain and Modified Live Virus (MLV), and currently licensed vaccines, fail to offer effective protection against the challenge of a heterogeneous strain, posing a great challenge to vaccine development. Thus, a new strategy for controlling this infectious disease is urgently needed.

Many natural compounds and herbal components have been confirmed to possess antiviral activities in Traditional Chinese Medicine (TCM). Natural herbal extracts contain many bioactive compounds featuring anti-inflammatory, antiviral and immune-regulatory activities, especially flavaspidic acid AB [12], glycyrrhizin [13] and platycodin D [14], which have been demonstrated to suppress PRRSV infection in vitro. The development of novel drugs might become an effective means to fight the global PRRS epidemic. However, the in vivo study of confirming TCM as a potential natural and effective anti-PPRSV agent was poorly described. Therefore, further studies in swine are necessary.

**Figure 1:**
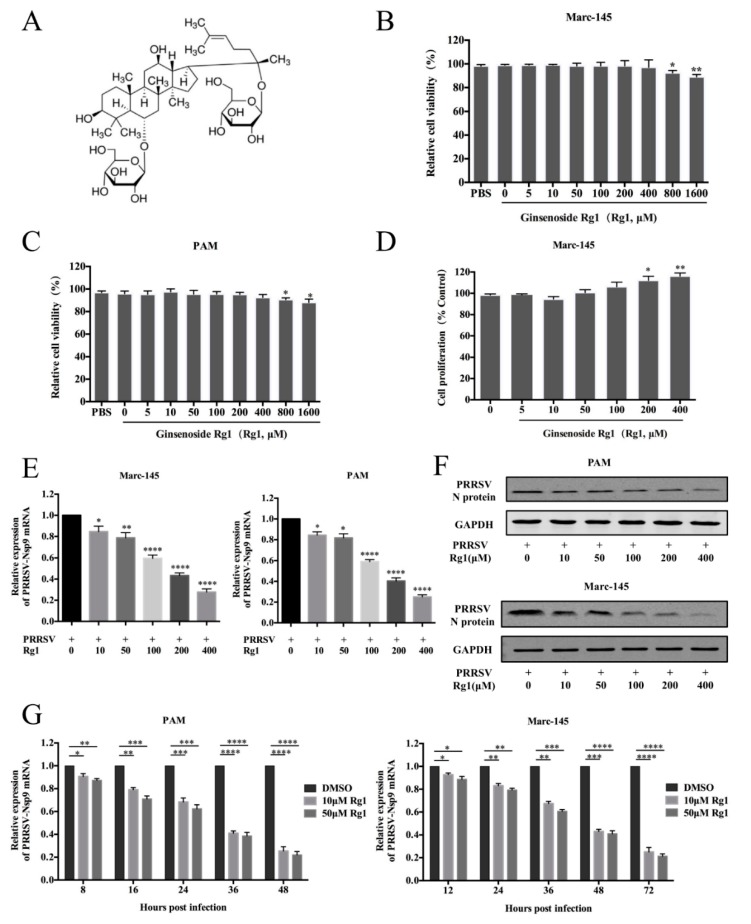
Cytotoxicity and anti-PRRSV activity of Rg1 in Marc-145 cells and PAMs. (**A**) The chemical structures of ginsenoside Rg1 (Rg1). (**B**,**C**) Cytotoxicity of Rg1 in Marc-145 cells (**B**) and PAMs (**C**) were analyzed by using the WST-1 assay. Results are showed as the relative cell viability of PAMs or Marc-145 cells cultured without Rg1 (set as 100%). (**D**) Rg1 affects the proliferation of Marc-145 cells. Cells were seeded and cultured without FBS for 12 h and the medium was replaced with DMEM contains 0, 5, 10, 50, 100, 200 and 400 μM Rg1 respectively. (**E**,**F**) PRRSV XH-GD (0.1 MOI) infected Marc-145 cells or PAMs for 1 h at 37 °C, and then cells were cultured in DMEM or RPMI 1640 supplemented with 2% FBS and indicated concentrations of Rg1. The samples were collected at 48 hpi to analyze PRRSV Nsp9 mRNA levels in different groups by RT-PCR (**E**). N protein expression levels in cells treated with different concentrations of Rg1 were detected by western blot (**F**). (**G**) Marc-145 cells and PAMs infected with PRRSV XH-GD (0.1 MOI) for 1 h at 37 °C and then cultured in fresh medium supplemented with 10 or 50 μM Rg1. The expression levels of PRRSV Nsp9 in Marc-145 cells and PAMs were detected by RT-PCR analysis at the indicated time points. Each data represents results of three independent experiments (means ± SD). Significant differences compared with the control group are denoted by * (*p* < 0.05), ** (*p* < 0.01), *** (*p* < 0.001) and **** (*p* < 0.0001).

Ginsenosides are biologically-active components of Panax ginseng and Panax notoginseng saponins that were widely used as a traditional herbal tonic in China for a thousand years, and ginsenoside Rg1 is a major bioactive component therein [15] (Figure 1A). A previous study demonstrates that Rg1 treatment enhances the immune responses induced by recombinant Toxoplasma gondii SAG1 antigen [16], and Rg1 could be used as an adjuvant to promote both T helper (Th) 1 and Th2 responses [17,18]. Treatment with Rg1 is found to significantly relieve the cellular inflammatory response in neurons [19] and reduce the expression of TNF-α, IL-1β and IL-6 in vivo [20]. However, the antiviral activity of Rg1 is poorly described. PRRSV infection results in the release of IL-1β, IL-6, IL-8 and TNF-α, and these pro-inflammatory cytokines contribute to the development of excessive systemic inflammatory reactions and pathological injury [6]. Thus, we set out to determine whether Rg1 has anti-PRRSV effects and possesses protective effects against viruses-induced injury.

Here we demonstrate that Rg1 exhibits an antiviral effect against a broad range of type 2 PRRSV in Marc-145 cells and PAMs, and Rg1 treatment reduces the mRNA levels of several pro-inflammatory cytokines triggered by PRRSV infection, and also inhibits the activation of the NF-κB signaling pathway. More importantly, piglets treated with Rg1 display decreased viremia, alleviated lung injury and increased survival rate after challenging with HP-PRRSV JXA1. Together, these data suggested that Rg1 might be a potential natural compound that could be applied in a PRRSV control strategy.

## 2. Materials and Methods

### 2.1. Cells and Virus

PRRSV strains, including the classical VR2332 strain (GenBank accession no. U87392.3; lineage 5.1), highly pathogenic XH-GD (GenBank accession no. EU624117; lineage 8.7) [21], and the NADC30-like strain HNLY (isolated in a sow with reproductive problem and saved in our lab; lineage 1), were saved in our lab. Highly pathogenic JXA1 (GenBank accession no. EF112445.1; lineage 8.7) was generously offered by Professor Tian [9].

All of the PRRSV strains were propagated and titrated in Marc-145 cells. Virus titers of each strain were calculated by using a Reed-Muench method.

### 2.2. Antibodies, Chemicals and Reagents

The mouse monoclonal antibodies against PRRSV N protein were purchased from MEDIAN Diagnostics (Korea). Rabbit monoclonal antibodies directed against Phospho-P65, Phospho-IκBα, and mouse monoclonal antibodies directed against P65 and IκBα were purchased from Cell Signaling Technology (Beverly, MA, USA). Goat anti-rabbit IgG antibody and goat anti-mouse IgG antibody were from LI-COR Biosciences (Lincoln, NE, USA). Glyceraldehyde 3-phosphate dehydrogenase (GAPDH) antibody was purchased from MBL Beijing Biotech (Beijing, China). Goat anti-Mouse IgG (H+L) Cross-Adsorbed Secondary Antibody (Alexa Fluor 594 and 488) were purchased from Thermo Fisher Scientific (Waltham, MA, USA).

Ginsenoside Rg1 (≥98.0%) was purchased from Chengdu Biopurify Phytochemicals (Chengdu, China). LPS (lipopolysaccharide from Escherichia coli 0111:B4) was purchased from Sigma-Aldrich (MA, USA). Rg1 was dissolved in dimethylsulfoxide (DMSO, Sigma-Aldrich) and diluted with DMEM before use. The final concentration of DMSO in the cell culture medium was less than 0.4%.

### 2.3. Quantitative Real-Time PCR

Total cellular RNA was extracted using a total RNA rapid extraction kit (Fastagen, Shanghai, China) according to the instructions, and 1 μg RNA of each sample was subsequently reverse transcribed to cDNA with a reverse transcription kit (TaKaRa, Dalian, China) according to the manuals. The acquired cDNA was then used as the template in a qPCR assay by using TB Green^®^ Premix Ex Taq™ II (Tli RNaseH Plus) or Premix Ex Taq™ (Probe q-PCR)(Takara Biomedical Technology, Beijing) in CFX96 Real-time polymerase chain reaction system (qPCR) (Bio-Rad, CA, USA). The abundance of individual gene mRNA transcripts in each sample was measured three times, and GAPDH mRNA was used as the endogenous loading control. The sequences of primers and probe are listed in Table 1. Relative mRNA expression of each target gene was calculated by the 2^−∆∆CT^ method.

### 2.4. Cell Proliferation and Cytotoxicity Assay

The cytotoxicity of Rg1 was analyzed by using the WST-1 Cell Proliferation and Cytotoxicity Assay Kit (Beyotime). Marc-145 cells (2 × 10^4^ cells per well) and PAMs (1 × 10^5^ cells per well) were seeded in 96-well plates and grown at 37 °C. Cells were incubated with medium supplemented with different concentrations of Rg1 and further incubated for 48 h. The absorbance of each well was read at 450 nm with a reference wavelength of 630 nm using a microplate reader (Thermo Fisher Scientific, MA, USA).

For the cell proliferation assay, Marc-145 cells (5 × 10^3^ cells per well) were seeded in 96-well plates and cultured without FBS at 37 °C for 12 h and then treated with indicated concentrations of Rg1. After 24 h of Rg1 incubation, the relative proliferation was evaluated using the WST-1 kit according to the instructions. The absorbance was read at 450 nm with a reference wavelength of 630 nm using a microplate reader (Thermo Fisher Scientific, MA, USA).

### 2.5. Antiviral Activity Assay

To analyze the effect of Rg1 on PRRSV infection, Marc-145 cells and PAMs were grown to 70%–80% confluence respectively in six-well plates. PRRSV strains (0.1 MOI) diluted in DMEM or RPMI 1640 were incubated with Marc-145 cells or PAMs at 37 °C for 1 h. The supernatants were removed, and the cells were washed twice with PBS. Then, fresh DMEM containing different concentrations of each compound was added and incubated at 37 °C in 5% CO_2_. After treatment, the cells and supernatants were collected at the indicated time points of post-infection. The supernatants were used to titrate the production of progeny virus, and the viral titers were defined and calculated as TCID_50_/mL [22]. The cell plates were washed with PBS and harvested for immunofluorescence assay (IFA), qRT-PCR, and western blotting analysis. The 50% effective concentrations (EC_50_) value (the concentration of Rg1 required to protect 50% cells from infection) of Rg1 against different type 2 PRRSV strains was determined as previously described [14], and calculated with the GraphPad Prism 7.0 software.

### 2.6. Immunofluorescence Staining

Marc-145 cells were grown on coverslips and then infected with PRRSV at 37 °C for 1 h. Cells were washed with PBS after infection and cultured with or without Rg1 (200 µM) for 24 h. Following Rg1 treatment, the cells were fixed with paraformaldehyde for 30 min at 4 °C. The fixed cells were permeabilized with 0.1% Triton X-100 in PBS for 5 min, blocked with 3% bovine serum albumin in PBS for 2 h, and endogenous proteins were directly stained with the respective antibodies. For IFA, mouse anti-N protein antibody and goat anti-mouse IgG Alexa Fluor 488 were used as primary and secondary antibodies respectively. Mouse monoclonal antibodies directed against P65 and goat anti-mouse IgG Alexa Fluor 594 were used in confocal assay. The nuclei were stained with 4′,6-diamidino-2-phenylindole (DAPI). Immunofluorescence was captured by Leica DMI 4000B fluorescence microscope (Leica, Wetzlar, Germany). The cover slips were mounted onto glass slides using PBS containing 50% glycerol. Confocal images were obtained using a laser scanning confocal microscope (Olympus, Japan).

### 2.7. Western Blotting

The total cell samples were washed twice with ice-cold PBS and then lysed in RIPA lysis buffer (Beyotime) with 1% phosphatase inhibitor cocktail (APExBIO, Houston, USA). The protein samples were resolved with sodium dodecyl sulfate–10% polyacrylamide gel electrophoresis (SDS-PAGE) and transferred to polyvinylidene difluoride (PVDF) membranes (Millipore, Billerica, MA, USA). The PVDF membranes were blocked with 5% BSA in Tris-buffered saline containing Tween 20 and then incubated with the primary antibodies. Goat anti-mouse or anti-rabbit IgG (LI-COR Biosciences) were used as the secondary antibodies. An Odyssey Infrared Imaging System (LICOR, CT, USA) was used to analyze the PVDF membranes.

### 2.8. Rg1 Treatment on PRRSV Life Cycle Assay

Marc-145 cells were grown to 70%–80% confluence in six-well plate at 37 °C in 5% CO_2_. Cells were infected with PRRSV. Four steps in PRRSV life cycle including attachment, internalization, replication and release were analyzed at different time-points after post-infection and the pre-treatment of Rg1 on PRRSV infection was also analyzed as previously described [14].

### 2.9. Animal Experiment

Thirty-two four-week old piglets, which were free of African swine fever virus (ASFV), PRRSV, antibody against PRRSV, pseudorabies virus (PRV) and swine influenza virus (SIV), were randomly divided into four groups with eight piglets in each group. For each group, five piglets were randomly selected and raised together as a sub-group used for collecting samples and recording clinical performance, morbidity and mortality, while the other three piglets were raised in another pigsty and euthanized at seven days post-challenge for pathological detection. The groups PRRSV and PRRSV+Rg1 were challenged intranasally (1 mL) and intramuscularly (1 mL) with the HP-PRRSV JXA1 strain (5 × 10^5^ TCID_50_), and the Mock group was inoculated in the same way with the same volume of DMEM and continued administration for 10 days, and then used as a negative control. At 12 h post infection, the group PRRSV+Rg1 was injected intramuscularly with Rg1 (10 mg/kg/day) and continued administration for 10 days. The PRRSV group was injected with 1 mL DMEM at 12 h post-infection and continued administration for 10 days, and then used as a challenge control group. The group Rg1 was injected with Rg1 (10 mg/kg/day) and continued administration for 10 days, being used as the drug control group. All of the piglets were planned to be monitored for 14 days after the challenge. Clinical signs and rectal temperatures of all of the groups were evaluated and scored daily. Blood samples of each piglet were obtained by venipuncture at 0, 2, 4, 7, 10 and 14 days post-infection (dpi) for the detection of the PRRSV viral load and anti-PRRSV antibody (Idexx PRRSV Elisa Kit, Idexx, US). The tissue sample of lung, thymus and lymph node were collected at 7 and 15 dpi. Tissue (1g) and serum (100 μL) samples were used to extract total RNA. RNA extraction and cDNA synthesis were performed as previous report [23]. Real-time quantification PCR was carried out on a CFX96TM real-time system (Bio-Rad, USA). Standard, serially-diluted PRRSV strain JXA1 (10^0^–10^7^ TCID_50_/mL) was used to generate a standard curve (slope = −3.717; R^2^ = 0.995) [24,25]. All pigs were euthanized for pathological detection at 15 dpi.

### 2.10. Clinical Performance and Gross Lesions of Lung

The clinical performance of all of the piglets after the HP-PRRSV JXA1 challenge were observed and scored daily, as described previously [26]. Briefly, the scores, ranged from 0 to 5, represent the severity of clinical symptoms, which including behavior, appetite and respiration. The total score for clinical performance was calculated by the sum of the evaluation index daily. The lung injury of each group was evaluated by necropsy at 7 dpi and 15 dpi. Macroscopic gross lesions of each lobe were determined as the percentage of lung with visible pneumonia and scored, while the obtained lung tissue of each group were used to perform histological pathology analysis as described [27].

### 2.11. Ethics Statement

Animal experiments in this study were approved by Laboratory Animal Committee of South China Agricultural University (No. 2019C001). All piglets were raised in the animal facility of SCAU, and all operations were in accordance with the animal ethics guidelines and approved protocols.

### 2.12. Statistical Analysis

All data of each assay represents at least two separate experiments and were determined in triplicate. The results collected from triplicate determinations were analyzed as the means ± standard deviations (SD). Data difference of each experiment was analyzed by one-way Analysis of Variance (ANOVA) followed by the Tukey’s t-test in GraphPad Prism 7.0 software (San Diego, CA). * *p* < 0.05, ** *p* < 0.01, *** *p* < 0.001 and **** *p* < 0.0001 were considered to be statistically significant at different levels.

## 3. Results

### 3.1. Ginsenoside Rg1 Treatment Supressed PRRSV Replication in Marc-145 Cells and PAMs

The cytotoxicity of ginsenoside Rg1 (Rg1) on Marc-145 cells and PAMs was analyzed by WST-1 assay. Rg1 does not impair Marc-145 and PAM cell viability at a concentration as high as 400 μM (Figure 1B,C). In the course of the experiment, we notice that Marc-145 cells cultured with Rg1 exhibit better cellular morphology under the serum deprivation. Therefore, whether Rg1 affects cell proliferation was analyzed in Marc-145 cells. The results indicate that Rg1 does not influence Marc-145 cell proliferation significantly at doses from 5 μM to 400 μM, and cell proliferation increases in 800 μM and 1600 μM (Figure 1D). These results suggest that Rg1 has minimal cytotoxicity on Marc-145 cells and PAMs within the tested doses.

To determine the anti-PRRSV activity of Rg1, Marc-145 cells and PAMs were infected with PRRSV XH-GD (0.1 MOI) for 1 h and then treated with indicated concentrations of Rg1 for 24 h. As shown in Figure 1E, treatment with Rg1 results in a significant dose-dependent reduction in PRRSV Nsp9 mRNA levels both in Marc-145 cells and PAMs. The expression level of N protein, evaluated by western blotting, decreases in proportion to the amount of Rg1 used in the treatment (Figure 1F). This result indicates that Rg1 treatment inhibits PRRSV replication, and 10 μM Rg1 could inhibit Nsp9 mRNA and N protein expression. Therefore, PRRSV inhibition kinetics by Rg1 in Marc-145 cells and PAMs are further analyzed at 10 μM and 50 μM. Nsp9 mRNA expression, representing the PRRSV replication rate in the treated groups, was compared to that in the DMSO-treated control (Figure 1G). The results reveal that the decrease in Nsp9 mRNA is more pronounced from 24 h.p.i. (hours post infection) to 48 h.p.i. in PAMs, and from 36 h.p.i. to 72 h.p.i. in Marc-145 cells. Collectively, these results suggest that Rg1 treatment suppresses PRRSV infection.

### 3.2. Rg1 Treatment Affect PRRSV Attachment, Replication and Release in Marc-145 Cells

**Figure 2:**
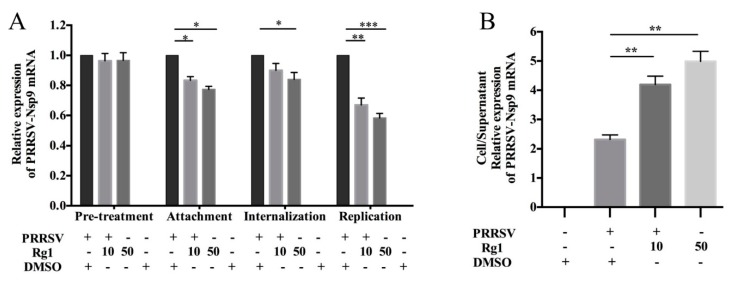
Inhibitory effects on the virus lifecycle of Rg1 in Marc-145 cells. In the Pre-treatment assay, Marc-145 cells were pretreated with DMEM supplemented with 10 or 50 μM Rg1 for 2 h, then cells were washed twice with PBS before being infected with type 2 PRRSV XH-GD (0.1 MOI), and then samples were collected at 48 h.p.i. For the attachment and internalization assay, Marc-145 cells were pre-cultivated at 4 °C for 1 h and then infected with virus (0.1 MOI) at 4 °C for 2 h. During virus attachment upon PRRSV infection, cells were cultured with DMEM or DMEM containing 10 or 50 μM Rg1 to analyze PRRSV Nsp9 mRNA level. Marc-145 cells were infected with XH-GD at 4 °C for 2 h and then cultured with or without Rg1 for 3 h at 37 °C. To avoid interference of other steps of viral lifecycle on replication assay, Marc-145 cells were infected with XH-GD for 6 h and then incubated with DMEM with or without 10 or 50 μM Rg1 at 37 °C, and samples were collected at 4 h.p.i. In all of the trials, GAPDH was used as a housekeeping gene for normalization, and cells treated with 0.4% DMSO was used as a reference control. (**A**) The effect of Rg1 on viral attachment, internalization, replication, and Rg1 pretreatment was analyzed by evaluating Nsp9 mRNA expression levels. (**B**) The effect of Rg1 on PRRSV release was detected by the ratio of Nsp9 RNA copy numbers in the supernatant and the cell lysate detected by qPCR. The analysis above was performed in triplicate. Statistical significance is denoted by * *p* < 0.05, ** *p* < 0.01, and *** *p* < 0.001.

To explore the effects of Rg1 upon the PRRSV life cycle, virus entry was analyzed in cell attachment (from 0–2 h.p.i.) and internalization assays (from 2–5 h.p.i.), virus replication was analyzed between 6–10 h.p.i. and virus assembly or release was determined after 12 h.p.i., based on the ratio of Nsp9 mRNA in infected cells and supernatant. The experimental strategy was as previously described [14]. The pre-treatment of Marc-145 with Rg1 does not reduce viral RNA levels, suggesting that Rg1 does not affect the susceptibility of Marc-145 cell to PRRSV significantly. For virus attachment, the experiment was designed to allow virus binding, but not cellular internalization, and the results show that the Nsp9 mRNA levels in cell lysates are reduced both in the 10 μM and 50 μM treatment groups (Figure 2A). Virus internalization was analyzed from 2–5 h.p.i. in the presence of Rg1, and the Nsp9 expression levels only decrease in the 50 μM group (Figure 2A). The inhibitory effect on PRRSV replication is significant in both the 10 μM and 50 μM groups (Figure 2A). As described previously, PRRSV progeny viruses are released 8 h.p.i. [13,28]. In addition, our results of PRRSV inhibition kinetics by Rg1 in Marc-145 cells indicate that viral mRNA significantly reduces from 12 h.p.i. to 72 h.p.i. (Figure 1G). Thus, we evaluated the virus release rate with Rg1 treatment at 12 h.p.i. Marc-145 cells were infected for 12 h at 37 °C, and the cells were then cultured in DMEM containing 10 μM or 50 μM Rg1 for another 2 h. The Nsp9 mRNA levels in infected cells and culture supernatants were quantified by RT-PCR, and the ratio of cell/supernatant Nsp9 corresponds to the virus progeny release rate. The results indicate that this PRRSV virus released from Marc-145 cells is remarkably suppressed by Rg1 treatment (Figure 2B).

### 3.3. The Anti-PRRSV Ability of Rg1 Treatment Was Effective in Marc-145 Cells and PAMs Infected with the HP-PRRSV, NADC30-Like and Classical Strains

**Figure 3:**
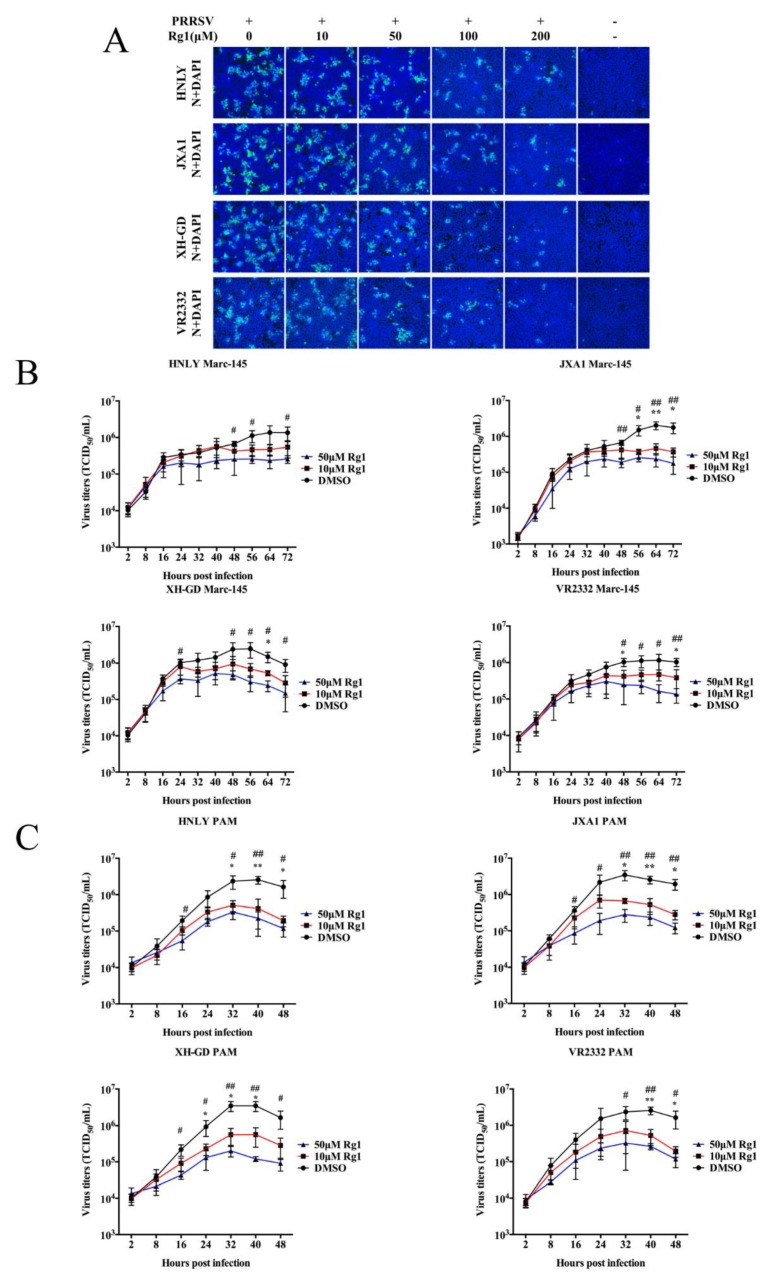
The antiviral activity of Rg1 against different lineages of type 2 PRRSV. (**A**) Antiviral activity of Rg1 against PRRSV strains (HP-PRRSV XH-GD and JXA1, classical VR2332 and NADC30-like strain HNLY) was determined in Marc-145 cells by IFA. Marc-145 cells were seeded in 12-well plates and infected with four type 2 PRRSV strain (0.1 MOI) respectively, and then incubated with DMEM supplemented with indicated concentration of Rg1. N protein was used as indicator of PRRSV infection, and the IFA detection of it was performed at 48 h.p.i. by using mouse anti-N protein antibody and goat anti-mouse IgG Alexa Fluor. Nuclei were counterstained with DAPI (blue). These images above represent three independent IFA trials with similar results. Magnification, 100 ×. (**B**,**C**) The inhibitory effect of Rg1 on PRRSV replication in Marc-145 cells (**B**), and PAMs (**C**). PRRSV replication was analyzed by virus growth curve. Marc-145 cells and PAMs were seeded in 6-well plates and infected with four PRRSV strain (0.1 MOI) for 1 h at 37 °C respectively and then cultured with DMEM or RPMI 1640 supplemented with 10 or 50 μM Rg1 or DMSO. Cell supernatants (200 μL) of each well were collected at indicated hours of post-infection. Growth assays for each group were performed in triplicate, and the resulting titers were determined as TCID_50_/_mL_ (the 50% tissue culture infectious dose per mL) and the data are shown as the means ± SD. T-test was applied to perform statistical analysis. Statistical significance between 10 μM Rg1 and DMSO is denoted by * *p* < 0.05 and ** *p* < 0.01, and significance between 50 μM Rg1 and DMSO is denoted by # *p* < 0.05 and ## *p* < 0.01.

In view of the high genetic variation among strains of type 2 PRRSV, we tested whether Rg1 possesses antiviral activity against broad lineages of strains. The antiviral effects of Rg1 against PRRSV strains, including JXA1 (HP-PRRSV; lineage 8.7), XH-GD (HP-PRRSV; lineage 8.7), VR2332 (classical strain; lineage 5.1) and HNLY (NADC30-like strain, lineage 1), in Marc-145 cells were examined by IFA and EC_50_. As shown in Figure 3A, the four lineages of PRRSV infection in Marc-145 cells were significantly inhibited by Rg1 in a dose-dependent manner. Therefore, EC_50_ of Rg1 against JXA1, XH-GD, HNLY and VR2332 infection in Marc-145 cells was calculated respectively. Results indicate that the values vary significantly, ranging from 55.05 to 94.21 μM among the four PRRSV strains by analyzing infection rate from IFA images (Table 2). To evaluate the inhibition effect of Rg1 on virus replication of four PRRSV, the growth curves of these strains were generated in Marc-145 cells. The results indicate that the inhibitory effect of Rg1 on the production of progeny virus is mainly seen at the plateau phase, and the antiviral activity is most obvious at 50 μM in all four PRRSV strains (Figure 3B). Moreover, the growth curves of the four PRRSV strains treated with Rg1 was further determined in PAMs, and the results display a similar decreasing trend (Figure 3C). These data indicate that Rg1 possesses an inhibitory effect on PRRSV infection and that this effect is observed in a broad range of PRRSV lineages.

### 3.4. Rg1 Treatment Significantly Reduced the Pro-Inflammatory Cytokine mRNA Levels Induced by PRRSV Infection in Both Marc-145 Cells and PAMs

**Figure 4:**
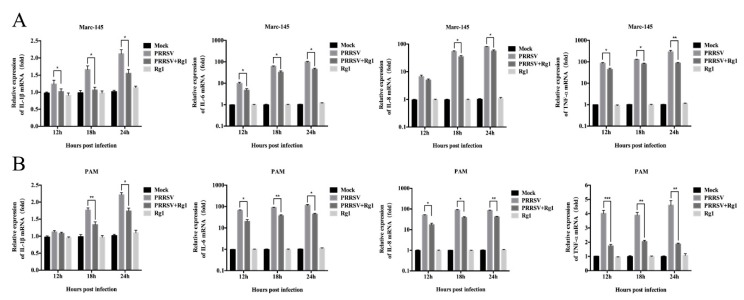
Rg1 suppresses inflammatory cytokines mRNA expression in infected PAMs and Marc-145 cells. (**A**) For PRRSV+Rg1 group, the XH-GD strain (0.1 MOI) infected Marc-145 cells for 1 h and then cultured in DMEM supplemented with Rg1 (200 μM), and cells infected with virus or treated with Rg1 (200 μM) were termed as PRRSV group and Rg1 group, respectively. Cells in the mock group were grown in DMEM containing 0.4% DMSO. Cell samples were collected to extract total RNA at 12, 18, and 24 h.p.i. The relative expression of IL-6, IL-8, IL-1β and TNF-α was analyzed by RT-PCR. GAPDH was used as internal control to normalize values. (**B**) PAMs were cultured with RPMI 1640 and treated as described above. The data of each trial represents three independent experiments and the values are shown as the means ± SD. T-test was applied to perform statistical analysis and the significance was indicated by asterisk in the graphs. Statistical significance is denoted by * *p* < 0.05, ** *p* < 0.01, and *** *p* < 0.001.

In view of the pro-inflammatory cytokines triggered by PRRSV, this contributes to its pathogenicity, and the role Rg1 plays in relieving inflammatory responses [19]. RT-PCR was used to determine whether Rg1 treatment alleviates the expression of pro-inflammatory factors, including IL-1β, IL-6, IL-8 and TNF-α, induced by PRRSV infection. The data indicates that Rg1 treatment significantly (*p* < 0.05) reduces the mRNA levels of pro-inflammatory factors increased by PRRSV infection both in Marc-145 cells and PAMs. In Marc-145 cells, mRNA level of IL-1β, IL-6 and TNF-α are significantly reduced by Rg1 from 12 to 24 h upon PRRSV infection, while, IL-8 is decreased by Rg1 at 18 and 24 h (Figure 4A). Further, pro-inflammatory cytokines assay was performed in PAMs and the results showed that IL-6, IL-8 and TNF-α triggered by PRRSV lowered by Rg1 pronouncedly from 12 to 24 h, while the reduction of mRNA level of IL-1β was seen at 18 and 24 h (Figure 4B). Although the inhibitory effect of Rg1 on each pro-inflammatory factor in Marc-145 cells and PAMs was not strictly consistent, the decline trend was both obvious at 18 h and 24 h. The results demonstrate that Rg1 could relieve the inflammatory responses caused by PRRSV infection via decreasing the mRNA expression of pro-inflammatory cytokines.

### 3.5. PRRSV-Infection Triggered NF-κB Activation Was Inhibited by Rg1 Treatment in Marc-145 Cells

**Figure 5:**
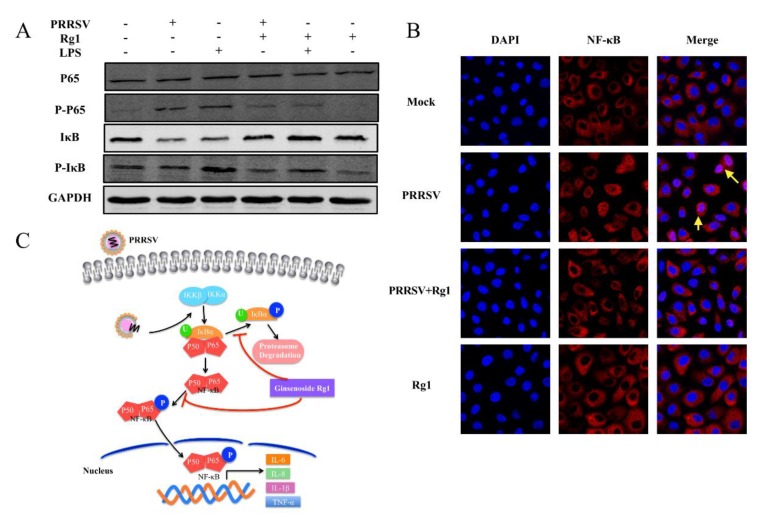
Rg1 inhibits the NF-κB pathway activated by PRRSV infection. (**A**) The expression and phosphorylation level of proteins involved in NF-κB pathway are analyzed in uninfected (Mock) and PRRSV XH-GD (0.1 MOI) infected Marc-145 cells treated with or without Rg1 (200 μM), samples were collected at 24 h.p.i. As a positive control, cells were cultured in DMEM and supplemented with LPS (2.5 μg/mL) at 37 °C for 6 h, and then the medium was changed to medium containing 0 or 200 μM Rg1 for 18 h. The western blotting data of each target protein represents three independent experiments with similar results. (**B**) Marc-145 cells were grown on glass cover slips and cultured in medium at 37 °C for 24 h, and then infected with PRRSV XH-GD (0.1 MOI). After virus infection, cells were incubated in fresh DMEM supplemented with or without 200 μM Rg1 for 24 h. Cells were washed twice with PBS and performed immunostaining by using anti-P65 antibody and red-fluorescent Alexa Fluor 594-conjugated goat anti-mouse IgG antibody. Nuclei were counterstained with DAPI. P65 protein deposited in the nucleus was indicated by yellow arrow. (**C**) Schematic model of Rg1 affect NF-κB signaling pathway upon PRRSV infection.

In the results above, Rg1 decreases the PRRSV-triggered mRNA level of pro-inflammatory cytokine. It is known that NF-κB is one of the key transcription factors regulating the production of pro-inflammatory factors, such as IL-6 and IL-8 [29]. PRRSV is proven to activate the NF-κB pathway to enhance viral replication [30]. Therefore, the effect of Rg1 treatment on PRRSV mediated NF-κB activation was analyzed by western blotting and immunofluorescence staining, and LPS stimulation, which activates NF-κB, was used as a control to show whether it associated with virus replication or indirectly through cellular response. We find that the increased phosphorylation of p65 induced by PRRSV infection or LPS is weakened by Rg1 treatment, and it also inhibits IκB degradation (Figure 5A). Although the phosphorylation of IκB-α, which is related to IκB degradation, was not significantly enhanced by PRRSV infection compared with mock, it was reduced by Rg1 treatment (Figure 5A). Moreover, the cellular localization of NF-κB was determined by p65 immunofluorescence staining (red) in Marc-145 cells, and the nuclei were stained with DAPI (blue) (Figure 5B). The results are consistent with the phosphorylation level of p65 by western blotting. In the Mock and Rg1 groups, P65 is mainly localized in cytoplasm and the phosphorylation level is lower. PRRSV triggers P65 phosphorylation to facilitate its translocation into nuclear, and this process is significantly reduced after Rg1 treatment. Taken together, these data indicate that Rg1 treatment decreases PRRSV-mediated NF-κB activation and IκB degradation, and a schematic of this event is presented in Figure 5C.

### 3.6. Rg1 Treatment Exhibits Antiviral Activity in Piglets

Given the antiviral activity of Rg1 against type 2 PRRSV that we analyzed in vitro, we wondered whether Rg1 could be used in antiviral therapy in piglets. HP-PRRSV JXA1 (EF112445) strain is a prototypical HP-PRRSV strain, characterized by discontinuous deletion of 30 amino acids in nonstructural protein 2, causes typical clinical symptom and pathological changes with high morbidity and mortality in piglets [9]. Here, we have evaluated the antiviral activity of Rg1 against JXA1 strain in vitro and calculated the EC_50_ of it (Table 2). Therefore, HP-PRRSV JXA1 was used to evaluate the anti-PRRSV effect of Rg1 in piglets.

#### 3.6.1. Clinical Signs and Mortality

**Figure 6:**
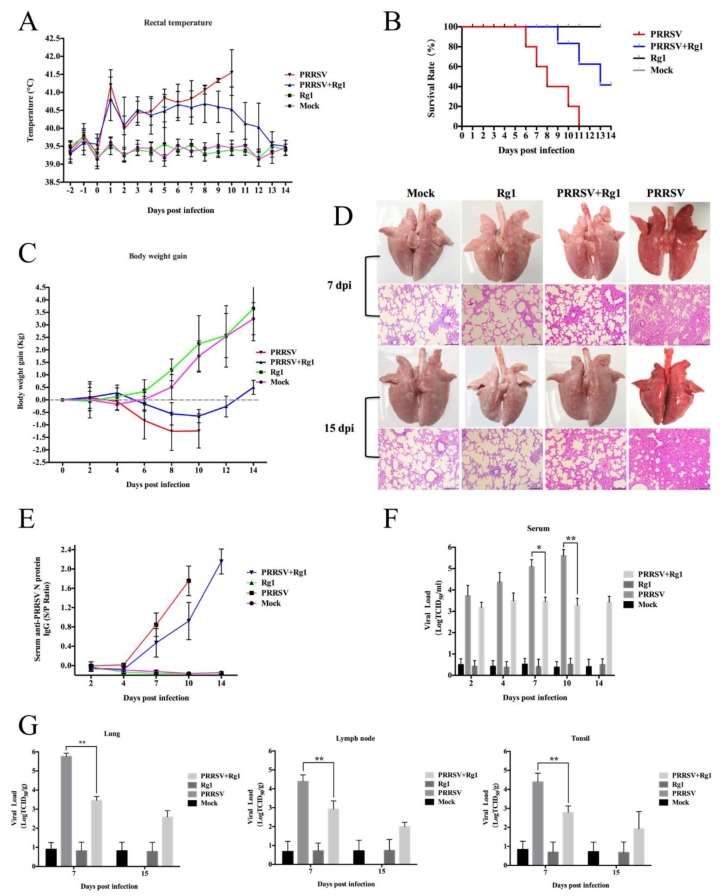
Rg1 exhibits anti-PRRSV activity in 4-week-piglet. (**A**) Daily rectal temperature of the pigs in the PRRSV, PRRSV+Rg1, Rg1 and mock groups. Rectal temperature reach or beyond 40 °C was defined as fever. (**B**) The mortality of each group was recorded daily and calculated as survival rate until 14 dpi. (**C**) The body weight gain of different groups during the experiment. (**D**) Severe lung lesions in the PRRSV group characterized by swelling, congestion, fibrosis, and inflammatory cell aggregates; however, in the PRRSV+Rg1 group, these index were moderate. Due to no piglet survived at 14 dpi in the challenge control group (PRRSV), the lung of the deceased pigs at 10 dpi was used to perform pathological analysis. (**E**) The anti-PRRSV antibody levels in serum at different time-points. The value of S/*p* ratio ≥ 0.4 was considered antibody positive. (**F**) The level of PRRSV mRNA in the serum was measured by real-time PCR. (**G**) The expression level of PRRSV mRNA level in lungs, lymph node and thymus was measured by qRT-PCR. Each tissue sample was measured three times, and the error bars represent the standard deviations of samples.

After being challenged with virulent HP-PRRSV JXA1, all piglets in the PRRSV group (piglets challenged with JXA1) displayed high rectal temperature (Figure 6A) and exhibited typical severe clinical signs, including in-appetence, lethargy, dyspnea, periocular and eyelid edema and hyperspasmia. Piglets treated with Rg1 after JXA1 challenge (PRRSV+Rg1 group) showed moderate anorexia and depression. Animals in Rg1 group (piglets only treated with Rg1) and mock group (piglets only treated with DMEM) behaved normally during the course of the experiment. The scores of the evaluation of clinical signs in the PRRSV+Rg1 group were significantly lower than PRRSV group at 7 d.p.i. (*p* < 0.05) (Table 3), a time-point of peak period of morbidity and mortality. All of the piglets displayed increased rectal temperature higher than 40 °C after challenge with HP-PRRSV JXA1, while piglets treated with Rg1 showed gradual descending since 8 dpi (Figure 6A). The survival rate of piglets in PRRSV group is 0% to 11 dpi, however, 40% animals in the PRRSV+Rg1 group (piglets treated with Rg1 after JXA1 challenge) survived to 14 dpi (Figure 6B). Since piglets showed different loss of appetite during infection course, the body weight of PRRSV and PRRSV+Rg1 displayed obviously drop after challenge, while the body weight of piglets in PRRSV+Rg1 displayed slower drop trend and begin to rise at 10 dpi (Figure 6C).

#### 3.6.2. Pathological Examination

As previously described, piglets infected with JXA1 began to die within 6–8 d.p.i. [9], and animals in the PRRSV group start to die at 6 d.p.i. in our study. Therefore, three living pigs of each group were euthanized for pathological examination at 7 d.p.i. to evaluate whether Rg1 treatment relieved lung injury.

The lungs of pigs in PRRSV+Rg1 group showed fewer pathological lesions and got significantly lower scores of macroscopic injury of lungs than those in the PRRSV group as shown in Table 3 (*p* < 0.05). Further histological examination of lung tissue showed that PRRSV+Rg1 group exhibited moderate interstitial pneumonia, while the PRRSV group were characterized by thickened alveolar walls, interstitial fibro-plastic proliferation and intensive mononuclear cell infiltration, which revealed the severe viral pneumonia (Figure 6D). At 15 dpi, all of the living pigs were euthanized for pathological examination. Due to no piglet survived at 14 dpi in the PRRSV group, the deceased pigs at 10 dpi of it were collected to perform pathological examination. The results displayed the same trend as 7 dpi. In the PRRSV+Rg1 group, the survived piglets at 15 dpi exhibited mild pneumonia compared with that of the mock group and Rg1 control group (Figure 6D).

#### 3.6.3. Viremia and Tissue Viral Load

Blood samples of the piglets were collected at 2, 4, 7, 10, 14 dpi to determine viral load and N protein antibody of PRRSV. And, the viral load in tissues, include lung, lymph node and thymus were determined by qRT-PCR as described previously [31]. Since all of the animals in the PRRSV group died at 11 d.p.i., there was no viremia and tissue viral load analysis on it at 14 or 15 h.p.i. The data revealed that piglets treated with Rg1 showed significantly lower viremia, indicated by antibody against PRRSV and viral load in serum, in the blood than those in PRRSV group at 7 (*p* < 0.05) and 10 dpi (*p* < 0.01) (Figure 6E,F). Meanwhile, PRRSV+Rg1 group displayed significantly reduced viral load in the lung, lymph node and thymus at 7 dpi (*p* < 0.01) (Figure 6G).

## 4. Discussion

PRRSV was identified in Europe in 1991 and emerged in United States in 1992 [32,33]. This virus is classified into European genotype (type 1) and North American genotype (type 2) [3], and the widespread outbreaks of PPRS in China are associated with constant evolution of viruses through high frequency of recombination and immune suppression events in recent decades. The protective immunity to PRRS elicited by current vaccines is effective only against homologous infections and exhibits partial protection from heterologous PRRSV. New antiviral therapeutic strategies became an urgent need. In the present study, we demonstrate that Rg1 suppressed broad lineages of type 2 PRRSV infection, including HP-PRRSV, classical strain and NADC30-like strains, both in Marc-145 cells and PAMs. It suggests that Rg1, as a natural herbal molecular, could be applied in broad-spectrum anti-PRRSV medicament.

PRRSV infection triggers the up-regulated release of IL-1β, IL-6, IL-8 and TNF-α [6,34,35]. These pro-inflammatory factors contribute to the stimulation of protective immune responses, while it also leads to the development of excessive systemic inflammatory reactions and causing inflammatory lesions [36]. Extensive studies have illustrated the potent anti-inflammatory effect of Rg1 treatment in various diseases by regulating inflammatory cytokine expression [37,38,39]. However, the anti-viral activity of Rg1 associated with modulating inflammatory response was poorly described. In our study, the results reveal that the expression level of several pro-inflammatory factors, including IL-1β, IL-6, IL-8 and TNF-α, is significantly reduced by Rg1 treatment in Marc-145 cell and PAMs upon PRRSV infection. It suggested that Rg1 moderate the PRRSV-induced inflammatory responses by decreasing mRNA expression of pro-inflammatory cytokines in vitro.

PRRSV infects host via membrane receptor mediated endocytosis process including virus attachment and binding, membrane fusion, and followed internalization [40,41]. Once the viral genome, single strand positive-sense RNA, is released into the cytoplasm, it substantially process translation to generate replication and transcription complex. Here, we performed analysis to determine whether Rg1 affect PRRSV lifecycle. The results indicated that pre-treatment of Rg1 could not affect virus host-cell tropism. Marc-145 cells treated with Rg1 during different period of infection process were analyzed respectively, the results showed virus attachment, internalization, and release were impaired (Figure 2). Meanwhile, the inhibitory effect or Rg1 on PRRSV replication stage was more obvious.

NF-κB, key transcription factor that regulates the activation of inflammatory cytokines, can be activated by virus infection, viral gene expression or by LPS stimulation [29], and it could be exploited by influenza viruses or type 1 HIV to sustain a high viral replication [42,43]. In view of the role of Rg1 played in suppressing pro-inflammatory factor expression triggered by PRRSV (Figure 4) and PRRSV-induced NF-κB activation facilitated its replication was demonstrated in previous report [30]. Therefore, we wondered Rg1 inhibited virus replication were associated with cellular process indirectly. In the present study, we identified that Rg1 alleviated PRRSV infection induced IκB degradation, phosphorylation level of p65 and p65 nuclear aggregation, important factors contribute to NF-κB activation, in Marc-145 cells (Figure 5). Moreover, our data indicated that Rg1 treatment suppressed NF-κB activity in LPS-treated Marc-145 cells (Figure 5), which was consistent with a previous study in mouse RAW 264.7 cells and macrophages [44]. This result suggested that the decreased NF-κB activation in Marc-145 cells, which treated with Rg1 upon PRRSV infection, was not due to a reduction in virus infection, it involved in the interaction between Rg1 and cellular process. Overall, the inhibitory effect on both IκB degradation and NF-κB nuclear translocation signaling contributes to the anti-PRRSV replication activity of Rg1. Possibly, it can be speculated that Rg1 mediated cellular process contributes to its anti-viral activity and it might be widely applied in other viral infection.

Recently, multiple Chinese traditional medicines were shown to possess anti-PRRSV ability in vitro [12,13,14]. However, the antiviral activities of these identified natural herbal extracts, active ingredients, compound or drugs have not been further evaluated in vivo. In present study, piglets challenged with HP-PRRSV JXA1 under Rg1 treatment showed increased survival rate, moderate pneumonia and lower serum and tissue viral loads compared to those in the PRRSV group (challenge control) (Figure 6). It suggests Rg1 might be a natural anti-PRRSV agent that could be considered as an adjuvant therapy in the pig herd production. Furthermore, it has been confirmed that ginseng extract inhibited virus infection including influenza virus and hepatitis C virus, and Rg1, which purified from the roots or stems of Panax notoginseng (PN) and Panax ginseng (PG), could also be used as an immunoadjuvant to improve immune responses [45,46]. In the current status, there are several Modified live Virus (MLV), which induced immune response did confer protection to vaccinated animals against homologous PRRSV challenge, licensed in various countries and extensively applied in pig farms [47,48]. However, existing evidence suggests that these MLV vaccines of type 1 or type 2 both stimulates limited humoral and cellular immunity and fail to against heterogeneous strain [49,50]. Therefore, it would be possible that ginseng extract used as a natural supplement in feeding management for disease prevention. Besides, it might be used in a combination with some direct-acting antivirals to achieve widely effective disease prevention or as immune-adjuvant of vaccination to reach immune enhancement. For the proper application of it, the pharmacokinetic data of ginseng extract in swine model needs further systematic study. Meanwhile, the optimum dosage and administration mode should be defined reasonably to avoid drug residues in meat production. Considering the economic benefit and most of the nutrient component were similar between PN and PG, PN might possess more potential value of application in the scaled raising of pigs.

In summary, our study demonstrated that ginsenoside Rg1 with low cytotoxicity and possess anti-PRRSV activity both in vitro and in piglets. And, it suppressed different lineages of type 2 PRRSV infections. These findings not only provide new insights into the molecular mechanism of Rg1 against PRRSV infection but also suggest a potential immune-modulatory and anti-viral agent in the control of the PRRS.

## Figures and Tables

**Table 1:** Sequences of the primers and probe used for Real-time polymerase chain reaction (PCR).

Name	Primer Sequence (5′-3′)
PRRSV Nsp9	F: CCTGCAATTGTCCGCTGGTTTG
	R: GACGACAGGCCACCTCTCTTAG
GAPDH	F: GCAAAGACTGAACCCACTAATT
	R: TTGCCTCTGTTGTTACTTGGAG
Marc-145 IL-6	F: GAGGCACTGGCAGAAAAC
	R: TGCAGGAACTGGATCAGGAC
PAM IL-6	F: CCTTCAGTCCAGTCGCCTTCTC
	R: CATCACCTTTGGCATCTTCTTC
Marc-145 IL-8	F: AGGACAAGAGCCAGGAAG
	R: CTGCACCTTCACACAGAGC
PAM IL-8	F: CACTGTGAAAATTCAGAAATCATTGT
	R: CTTCACAAATACCTGCACAACC
Marc-145 TNF-α	F: TCTGTCTGCTGCACTTTGGAGTG
	R: TTGAGGGTTTGCTACAACATGG
PAM TNFα	F: TGGTGGTGCCGACAGATGG
	R: GGCTGATGGTGTGAGTGAGG
Marc-145 IL-1β	F: GGAAGACAAATTGCATGG
	R: CCCAACTGGTACATCAGC
PAM IL-1β	F: ACCTGGACCTTGGTTCTCTG
	R: CATCTGCCTGATGCTCTTG
Probe JXA1 Nsp9	ACTGCTGCCACGACTTACTGGTCACGCAGT

F: forward primer; R: reverse primer.

**Table 2:** Inhibitory activity of Ginsenoside Rg1 against PRRSV infection in MARC-145 cells.

	PRRSV Strain			
	XH-GD	JXA1	HNLY	VR2332
EC_50_ (μM) ^a^	75.05 ± 13.52	71.33 ± 13.43	94.21 ± 8.27	55.05 ± 4.535

^a^ the concentration required to protect 50% cells from PRRSV infection by counting cells from IFA images.

**Table 3:** The scores of clinical signs and lung lesions of the different groups at 7 d.p.i.^a^.

Groups	Clinical Signs Scores (±S.D.) ^b^	Lung Lesions Scores (±S.D.) ^c^
PRRSV (Challenge control)	13.270 ± 1.8931^1^	76.0 ± 18.171^1^
PRRSV+Rg1	8.807 ± 2.5302^1^	34.0 ± 11.402^1^
Mock	0^2^	0^2^
Rg1	0^2^	0^2^

^a^ Values followed by letters 1 represents significant difference (*p* < 0.05) between PRRSV and PRRSV+Rg1; letter 2 represents significant difference (*p* < 0.05) between Mock/Rg1 and PRRSV/PRRSV+Rg1. ^b^ The clinical sign score was calculated by sum of behavior performance, appetite, respiration according to the extent of severity. ^c^ Analyzing the percentage of the macroscopic lesion features of pneumonia in entire lung.
